# Classification of coffee leaf nutrient deficiencies using hybrid feature aggregation with hierarchical localized attention and MobileNet

**DOI:** 10.3389/frai.2026.1784938

**Published:** 2026-04-28

**Authors:** S. Alden Jenish, R. Karthik, S. Keerthana, G. Sudhakaran

**Affiliations:** 1 School of Electronics Engineering, Vellore Institute of Technology, Chennai, India; 2 Centre for Cyber-Physical Systems, Vellore Institute of Technology, Chennai, India

**Keywords:** coffee leaves, collaborative attention, convolutional neural network, deep learning, nutrient deficiency

## Abstract

**Objectives:**

Nutritional deficiency in coffee is a major problem that compromises plant health, crop yield, and bean quality, directly threatening the economies of coffee-dependent regions. Traditional detection methods are primarily manual, time-consuming, and relied upon expert availability.

**Methods:**

This study introduces a novel Deep Learning (DL)-based dual-track architecture designed for the efficient classification of nutritional deficiencies in coffee leaf. The first track utilizes a MobileNetV3 backbone integrated with a Multi-Convolutional Shape-Aware Kernel (MCSK) block to capture spatially adaptive features from leaf textures and vein patterns. The second track employs a Hierarchical Shuffled Group Attention Network (HSGAN), utilizing Efficient Channel Attention (ECA) and Local Group Attention (LGA) modules to balance fine-grained local variations with broad spatial dependencies. Finally, a Multidimensional Collaborative Attention (MCA) mechanism is applied to the fused features to enhance cross-channel interactions and feature extraction.

**Results:**

The proposed model was evaluated using the CoLeaf dataset, where it achieved an accuracy score of 96.04%. This performance demonstrates an improvement over existing research and current state-of-the-art models, highlighting the architecture’s ability to identify complex nutrient-related patterns in coffee leaves.

**Conclusion:**

The performance of the proposed DL approach offer a solution for the automated monitoring of coffee plants. By providing a reliable alternative to manual inspection, this method presents the potential to help coffee production and support the agricultural regions worldwide.

## 1 Introduction

Coffee is produced from the seeds of the plants belonging to the Coffea genus, specifically *Coffea arabica* and *Coffea canephora*. Countries such as Brazil, Vietnam, and Colombia depend on coffee production as an important agricultural economic sector. The coffee plant’s health is vital for sustaining its yield, productivity, and quality during production. However, nutritional deficiencies present a significant risk to these plants. Deficiency in macro- and micronutrients results in visual symptoms on leaves, including chlorosis, necrosis, and stunting, that reduce the plant’s capacity for photosynthesis and crop production. They can damage these plants by weakening them and increasing their susceptibility to environmental stressors. This results in potential yield loss and threatens the sustainability of coffee production.

Traditional approaches for diagnosing nutrient deficiency symptoms are primarily based on the visual inspection of the agronomists or through laboratory analyses of the leaf samples (Barbedo, 2018). Visual inspections can be subjective and often depend on the agronomist diagnosing the more obvious symptoms, which are often associated with visible leaf curling and chlorosis. Many of the symptoms can lead to ambiguous diagnoses that overlap with those for pests or diseases (Ferentinos, 2018). This makes the accurate detection of these deficiencies difficult and increases the error rates. Additionally, the laboratory approaches are often costly, require multiple batches of procedural steps, and often have considerable time delays before results are available to agronomists and farmers. Recent advances in Computer-Aided Diagnosis (CAD) and Artificial Intelligence (AI), specifically Machine Learning (ML) and DL-based systems, have transformed agricultural diagnostic capabilities and can help agronomists automate the detection of nutrient deficiencies in coffee leaves (LeCun et al., 2015). Automating leaf image processing using ML or DL algorithms for deficiency symptoms is more efficient than the traditional systems and helps identify patterns based on color, texture, or structure (Kamilaris and Prenafeta-Boldú, 2018). Further, these systems can process large data provide to find reliable results and analyses that are accurate, allowing the farmer to take necessary treatments in nutrient management. Additionally, these methods are also non-invasive, as manual or human inspections require physical sampling in some cases. These methods do not affect the plant leaves and can potentially diagnose without degrading, creating a significant advantage for continued in-field processing and monitoring. Furthermore, the data sets and algorithms are applicable in field situations and unaffected by any growth stage. Thus, automated systems help provide more reliable, scalable agricultural processes while reducing unjustified losses in yield, contributing to sustainable coffee production (Mohanty et al., 2016). Early and accurate detection prevents excessive or incorrect fertilizer usage, reducing resource waste, minimizing environmental runoff, and lowering production costs. By supporting data-driven nutrient management, such systems contribute to more efficient input utilization and improved long-term soil health. Further refinements in the field will produce and employ more sustainable diagnostic systems to meet the important and timely objectives associated with precision agriculture.

With the increasing use of ML and DL in agriculture, solutions are emerging to satisfy the need for a reliable and efficient method of detecting nutrient deficiency. These approaches provide uniform predictions despite environmental variations. Since human visual inspections can vary and laboratory tests require considerable time, machine learning and deep learning provide a more reliable and efficient approach for achieving accurate results. The major contributions of this research include

- A dual-track architecture combining MobileNetV3 enhanced with MCSK blocks and the customized HSGAN track, enabling comprehensive capture of both global and fine-grained visual patterns in nutrient-deficient leaves.
- The customized HSGAN track incorporates attention mechanisms to efficiently extract local features while preserving spatial relationships, improving discrimination among visually similar deficiency classes.
- Multi-stage feature fusion and Spatial Pyramid Pooling (SPP) to ensure efficient representation across scales, capturing both contextual structure and localized symptoms for precise classification.

## 2 Related works

Extensive research has focused on developing automated systems capable of diagnosing nutrient deficiencies in plant leaves across various species. This section presents a literature review of several automated detection systems based on ML and DL to classify nutrient deficiencies in plants. The ML approach consists of feature extraction from leaf images, such as color, texture, and shape, that can show subtle signs of some nutrient imbalances. The characteristics are then analyzed and identified as developing signs in the leaf, improving the detection of potential deficiencies. This approach prioritizes factors related to surface texture and discoloration, which will be beneficial in the early detection of nutrient-related health issues. Nonetheless, DL approaches have considerable improvements over classical ML procedures for deficiency detection and classification. In contrast to ML-based methods, where features are manually extracted from data, in DL complex feature representation is learned directly from the raw input data.

Jin et al. (2022) employed a portable spectrometer to obtain reflected leaf spectra from freshly harvested leaf samples. This study utilized a swarm intelligence algorithm to discover the optimal wavelengths and presented performance metrics for different ML models trained using the spectral features that were extracted. Pandey et al. (2023) utilized partial least squares regression models to predict nutrient concentrations in plants, using spectral data. The expected outputs of the model were nutrient concentrations, which were utilized to determine deficiency by comparing the predicted nutrient concentrations to threshold responses and providing summaries in both categorical and continuous formats. Xu et al. (2023) tackled the problem of personalized nutrient diagnostics by using digital image processing and ML to identify potassium-deficient leaves. Different ML algorithms were applied to accurately classify potassium deficiency in leaves. Lu et al. (2023) developed methods using Gray-Level Co-occurrence Matrix (GLCM), Local Binary Pattern (LBP), and others for the extraction of features from plant leaves. Both traditional ML and DL models were developed, and the performance analysis of both types of models was discussed. Rahadiyan et al. employed feature aggregation to analyze leaves that showed nutrient deficiency. The study utilized LBP and GLCM methods to extract texture features (Rahadiyan et al., 2023). The authors stated that combining features was not always guaranteed to improve performance when classifying leaves and should be done with respect to optimal classification accuracy. Premi (2024) applied adaptive histogram equalization to improve the image quality of the samples. The features of texture, shape, and color were extracted, and then they were used to train a hybrid CNN model for classification. Kamelia et al. (2024) showed an application of Artificial Neural Networks (ANN) and Support Vector Machines (SVM) for the classification of nutrient deficiency in *Citrus reticulata* leaves. Qur'ania et al. (2020) employed an ANN model to investigate nutrient deficiencies from RGB images taken at different stages of growth. The preprocessing involved segmenting, feature extraction, using color histograms in RGB, and some statistical descriptors.

Jeong et al. (2025) devised an object detection method employing the YOLO model to detect nitrogen, phosphorus, and potassium deficiencies in leaves. The dataset was generated over an extended time frame in which the plant was grown under nutrient-deficient conditions. The object detection model was developed for use in real-time assessments in an agricultural field setting. On the other hand, Jose et al. (2021) developed a multi-label classification model based on ANN to detect six macronutrient deficiencies. Their multi-label approach involved preprocessing the input images through hue-based segmentation techniques, while feature extraction used the GLCM. The model was utilized in a mobile application for the real-time assessment of nutrient status and grading of fertilizer recommendations.

Patel et al. (2025) evaluated the effectiveness of pre-trained image classification models, along with CNN and combined SVM and CNN. Venkatesh and Naik (2024) presented a modified VGG16 model for nutrient deficiency detection and severity of plant leaves. This model detects deficiency of nutrients from self-collected images from experimental fields for two seasons and is shown to be effective in predicting crop yield. Recent literature has shown that coupling DL networks with optimization algorithms plays a vital role in enhancing stable convergence and improving classification performance (Anupama et al., 2022; Mothilal and Kumar, 2025; Vijayakumar, 2025; Mothilal and Kumar, 2024). Hariri and Avşar (2022) presented a sequential CNN model adjusted with the Particle Swarm Optimization (PSO) algorithm to assess calcium deficiency with real-time restraints. The study optimized the hyperparameters using PSO and compared performance over eight benchmark transfer learning models. Song et al. (2023) employed shallow feature extraction from images and deep features using the ConvNeXt-Base network. The fused shallow and deep features are trained using three novel algorithms to accurately identify nutrient-deficient pear leaves. Kolhar et al. (2024) presented nutrient deficiency classification analysis by implementing models such as Xception, the vision transformer, and the multi-layer perceptron mixer model. Ramos-Ospina et al. (2024) presented the performance analysis of various deep transfer learning models for phosphorus nutrition state classification in plant leaves. The study also incorporated several preprocessing and augmentation techniques to balance the dataset for more accurate performance.

Shadrach et al. (2023) introduced a deep transfer learning framework enhanced by the optimization ring toss game algorithm for the early identification of nutrient deficiencies. It also implemented Gabor filters and SqueezeNet for image preprocessing and feature extraction, respectively. Muthusamy and Ramu (2024) implemented an ensemble-based CNN model to identify micronutrient deficiencies in crops via images of the leaves. The study proposed the use of six pretrained DL models and adjusted the hyperparameters of their top dense layers. The classification was implemented via an averaging strategy for three binary models. Sharma et al. (2022) also employed an ensemble-based architecture that used transfer learning to identify nutrient deficiencies. The study implemented an ensemble pruning process using a ranking process where models were ordered based on model feature criteria, which reduced the complexity of the ensemble and increased performance. Sathyavani et al. (2021) introduced an image acquisition-based detection model utilizing the Internet of Things (IoT) approach to identify nutrient deficiency in crops. The study utilized a deep DenseNet-BC CNN for the classification of bug-identified images. Espejo-Garcia et al. (2022) presented the study of architectures such as EfficientNet, MobileNetV3-Large, and DenseNet for nutrient deficiency classification. The study highlighted the impact of the architectures on quality and efficiency, with the demonstration of their efficacy for diagnosing nutrient deficiencies. Tuesta-Monteza et al. (2023a) utilized the ResNet50 architecture to classify nutritional deficiencies in coffee leaves. Bera et al. (2024) implemented a mixed architecture based on a Graph Convolutional Network (GCN) and a custom CNN to increase and improve the classification of nutritional deficiencies in plants. The study employed embedding region-based pooling of fixed sizes, combined with multi-scale spatial pyramid pooling, to progressively enhance the aggregation of features. Sai Charan et al. (2025) introduced a dual-track architecture based on DenseNet and a custom CNN network. The local and global feature representations were concatenated to classify the samples effectively. In summary, these approaches provide exciting observations into the possibilities of ML and DL systems as diagnostic tools to identify nutrient deficiencies in plants.

### 2.1 Research gaps

This study aims to address gaps in the classification of leaf nutrient deficiencies. The key research gaps identified are

1. Most of the models utilized are basic pretrained DL models with no specific capacity to capture the small visual traits related to nutrient deficiency in coffee leaves. The unspecialized nature of these models limits their utility when dealing with the accurate classification of small details in texture, color, or size.
2. For nearly all classification approaches, coffee nutrient deficiency classes are treated as qualitatively separate and typically mutually exclusive observations, disregarding any visually similar, overlapping symptom classes. This can hinder the model’s learning and impact overall classification rates, particularly for nutrient deficiency classes that have a strong similarity in the visual presentation of attributes.
3. The research literature has done little exploration of hierarchical or multi-scale architectures that can capture both global structural aspects of the leaf and fine-grained and identifiable local patterns of leaf symptoms. This makes the models limited in feature representations and may incorrectly normalize the contextual and precise details.

### 2.2 Research contributions

This study proposes a novel dual-track architecture for classifying the type of nutritional deficiencies in coffee leaves. The contributions reported in this study are

1. The proposed approach is a dual-track CNN architecture using MobileNetV3 enhanced with the novel Multi Convolutional Shape-aware Kernel (MCSK) block in the first track and the Hierarchical Shuffled Group Attention Network (HSGAN) in the second track. The proposed MCSK and HSGAN help improve feature representation through structured integration of attention-guided operations inspired by the existing literature. It facilitates the capturing of high-level structural clues while considering subtle local differences between the nutrient-deficient leaves and alleviating some of the limitations of generic models.
2. The HSGAN track includes Local Group Attention (LGA) and Channel Shuffle (CS) modules, which allow attention to the specific details of textures and areas of overlapping symptoms while reducing misclassification errors due to the visual similarities in nutrient-deficient classes.
3. The model’s architecture incorporates multi-scale and hierarchical pathways in its convolution operations and helps the model learn both the overall leaf structures and the specific patterns of symptoms. This hierarchical feature representation ensures context awareness and fine detailing.

## 3 Proposed network

The proposed network employed a dual-path architecture designed specifically for efficient and accurate image classification of nutrient-deficient coffee leaves. The first track utilizes a modified MobileNetV3 as its backbone combined with an MCSK block, providing a better way for the network to capture spatially varying, shape-aware features from textures and leaf vein patterns. The second track utilizes a fully customized CNN, which has Efficient Channel Attention (ECA), LGA, and CS. This second track emphasizes extracting complementary fine-grained features for relationships between channels and the spatial correlations through the HSGAN. This dual-track design helps explicitly decouple global and fine-grained local symptom modeling. Nutrient deficiencies in coffee leaves present both macroscopic patterns, such as discoloration, and microscopic variations, such as texture irregularities. A single backbone model may limit its discrimination ability among visually similar classes. The dual-track architecture helps ensure both global contextual cues and localized discriminative patterns are learned to improve performance.

**Figure 1:**
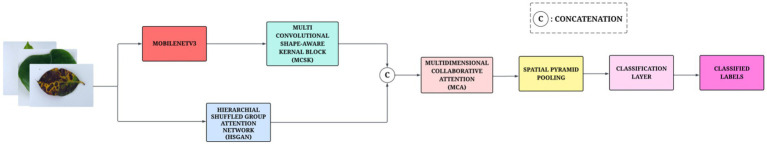
Overall architecture design of the proposed dual-track DL system for coffee leaf nutrient deficiency classification.

Both tracks work in parallel, and their output is fused by concatenating the features. This process maintains broad contextual indicators while still representing detailed local information, thus enhancing the favorable representation of nutrient-specific attributes in coffee leaves. For improved feature representation, the fused features are refined through a Multidimensional Collaborative Attention (MCA) block before pooling. The MCA module models attention shares across the channel, height, and width, unlike regular attention methods that look at each of these dimensions individually. This shared approach provides better feature representation by allowing it to be more sensitive to fine-layer textural differences, edge formations, and occlusions present in nutrient-deficient leaf images. After the MCA refinement, an SPP layer is added to aggregate features across scale, providing greater efficacy to size and shape changes. An overview of the proposed system can be seen in Figure 1.

### 3.1 MobileNetV3 with multi-convolutional shape-aware kernel block

The proposed system leverages the MobileNetV3 architecture, an efficient backbone network that balances communication cost and representation ability, and incorporates lightweight architectural components like depthwise separable convolutions, inverted residual bottlenecks, and Squeeze-and-Excitation (SE) attention blocks to enhance efficiency. MobileNetV3 utilizes hard-swish activation layers as well as search-based optimizations to further generally increase efficiency. However, MobileNetV3’s feature extraction strategies specialize in local depthwise convolutions and channel attention, often limiting the amount of rich spatial dependency, shape-aware structures, and multi-branch contextual features. To address this, the study incorporates a novel Multi-Convolutional Shape-Aware Kernel (MCSK) block after the MobileNetV3 backbone that extracts additional richness in feature extraction.

The MCSK block improves MobileNetV3’s representational ability by combining two independent paths for complementary feature extraction that operate in parallel to capture different types of information. The first path uses a shape convolution that includes geometric information in the convolutional process (Cao et al., 2021). It takes an input patch $P\in R^{K_{h}\times K_{w}\times C_{\text{in}}},$ and breaks it into two complementary components: a base component that captures average intensity and a shape component that captures relative variation, as represented by Equations 1 and 2:

$$\underset{B}{P}=m(P)$$ (1)

$$\underset{S}{P}=P-m(P)$$ (2)

Where, m(P) is the mean function over the spatial kernel dimensions. Unlike vanilla convolution, it introduces two learnable weights, $W_{b}$ for the base component and $W_{s}$ for the shape component, which are combined before standard convolution as per Equation 3:

$$F=\text{Conv}(K,(W_{B}⊙P_{B})+(W_{S}*P_{S}))$$ (3)

Where, ⊙ and ∗ represent denote base-product and shape-product operations, respectively. Finally, the shape-aware patch is reconstructed using Equation 4:

$$P_{\mathrm{BS}}=P_{B}+P_{S}$$ (4)

where, $P_{\mathrm{BS}}$ is the shape-aware patch, which is passed through the convolution kernel to generate enriched feature maps.

The second path employs augmented convolution, which is a hybrid method that merges standard convolution with self-attention (Bello et al., 2019). Convolutional operations have certain issues, especially that their spatially local receptive fields might limit them from modeling long-range dependencies. Augmented convolution addresses this issue by utilizing a separate Multi-Head Attention (MHA) branch arranged parallel to the traditional convolution path. The results of both branches are merged, allowing the operator to model local patterns while simultaneously modeling higher-order contextual relationships in a single computational path. Shape and augmented convolution combine to form a twin-pathway architecture that learns complementary spatial representations. During this process, a lightweight Ghost Module (GM) operates on the merged feature maps, maximizing computational efficiency while providing accurate spatial representations.

**Figure 2:**
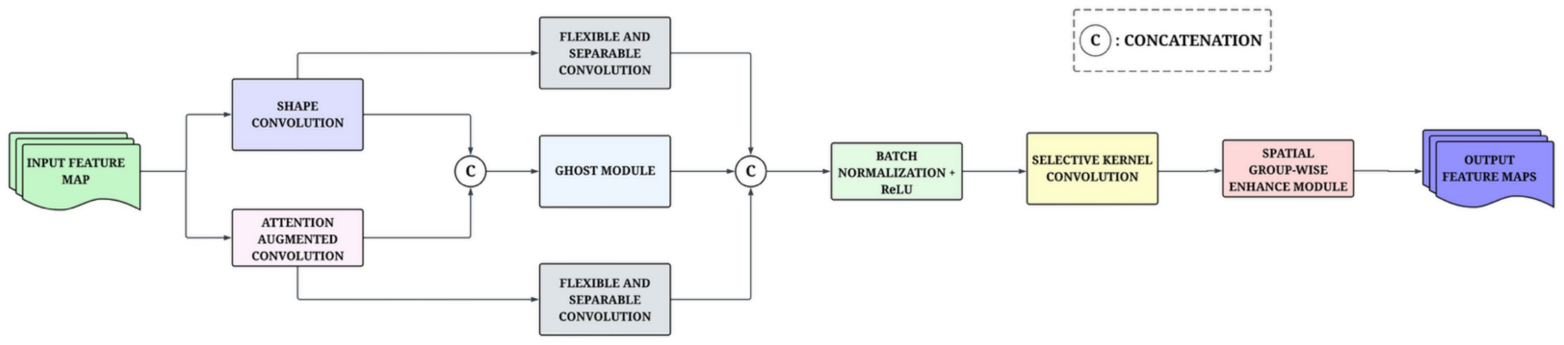
Architecture of the MCSK block to enhance shape and structural feature representation of MobileNetV3.

In addition, both complementary methods utilize a flexible and separable convolutional block to gain the precision in localized features (Zhu et al., 2023). The method adaptively separates them into two groups: representative feature maps processed with standard convolutional layers for intrinsic information extraction and then redundant feature maps processed by a lightweight convolutional layer for additional fine-grained detail extraction. The module also combined the CS, batch normalization, and shortcut residual connections to stabilize dynamic training and gradient propagation. The resulting feature maps extracted from the two concatenated branches are concatenated and then processed as a Selective Kernel (SK) convolution layer mechanism (Gao et al., 2021). The convolution is dynamic, allowing for receptive field adjustments to each convolution layer and overcoming the fixed kernel constraint of CNNs. This SK mechanism does not rely on a single kernel; instead, it employs a multi-branch structure that incorporates multiple kernel sizes, enabling the network to analyze features recursively across various receptive fields. Subsequently, a module calculating importance weights corresponding to each branch, which allows the model to focus on the most relevant kernel outputs based on the context, is computed as an attention module. The adaptive kernel selection can leverage both exact as well as contextual wide-level representations, providing additional indicative value to representations nested within a spectrum of complexities of data. This hybrid fusion process will contribute to the modeling of the global context as well as the representation of the detailed local patterns. The fused feature map is then processed by the Spatial Group-wise Enhance (SGE) module, which computes attention at the group level for each spatial location (Li et al., 2023). The module calculates an attention mask based on measuring the similarity of global and local features, enhancing the most informative spatial regions while suppressing extraneous or redundant responses. The MCSK enhances the MobileNetV3’s sensitivity to shape-dependent and spatially variant patterns commonly observed in nutrient deficiency classes. Unlike traditional depthwise convolutions that primarily focus on channel-wise efficiency, the MCSK explicitly models relative spatial deviations and geometric structures. This shape-aware enhancement helps reduce the confusion between nutrient deficiencies that exhibit similar color distributions but differ subtly in leaf structure. A structural view of the MCSK block is provided in Figure 2.

### 3.2 Hierarchical shuffled group attention network

The HSGAN serves as the second track of feature extraction within the proposed architecture. The network builds sophisticated feature representations over a series of convolutional and attention-based modules. The network starts with the push-pull convolution (Bennabhaktula et al., 2024). The push-pull convolution module deals with efficiency versus noise while allowing the network to reinforce structural information, such as edges and contours. The module outputs consider the rectified difference between a standard convolution (push) compared to an inhibitory convolution (pull) applied to the image. The push kernel serves the function of a convolutional filter, and the pull kernel behaves as a countering convolution that is found in its contrasting pattern. This process incorporates nonlinearity and inhibition that enhances feature representation. The fundamental framework of the architecture is a series of stacked blocks that are ranked hierarchically, with deeper blocks containing larger channel dimensions. Each block processes inputs in two convolution pipelines running in parallel, with the first pipeline emphasizing effective local feature extraction. It begins with a pointwise convolution that alters the channel dimensions, followed by a depthwise separable convolution that retains spatial information while optimizing using computations. In the second pipeline, global context dependence is introduced on top of local context. The process begins with a wider 5 × 5 convolution that accelerates the receptive field. This is followed by an LGA module that divides the channels into subgroups of branches and applies attention to each subgroup (Lu et al., 2025). The hierarchical arrangement of the HSGAN is tailored to improve the performance of the model against overlapping visual symptoms among nutrient deficiency classes. While global and shape cues of the coffee leaves may be similar across deficiencies, local and spatial distributions often vary. The LGA enables the network to focus on these localized variations by selectively weighting spatial regions within channel groups. The ECA module further refines it by amplifying the most discriminative channels, allowing HSGAN to prioritize subtle patterns.

**Figure 3:**
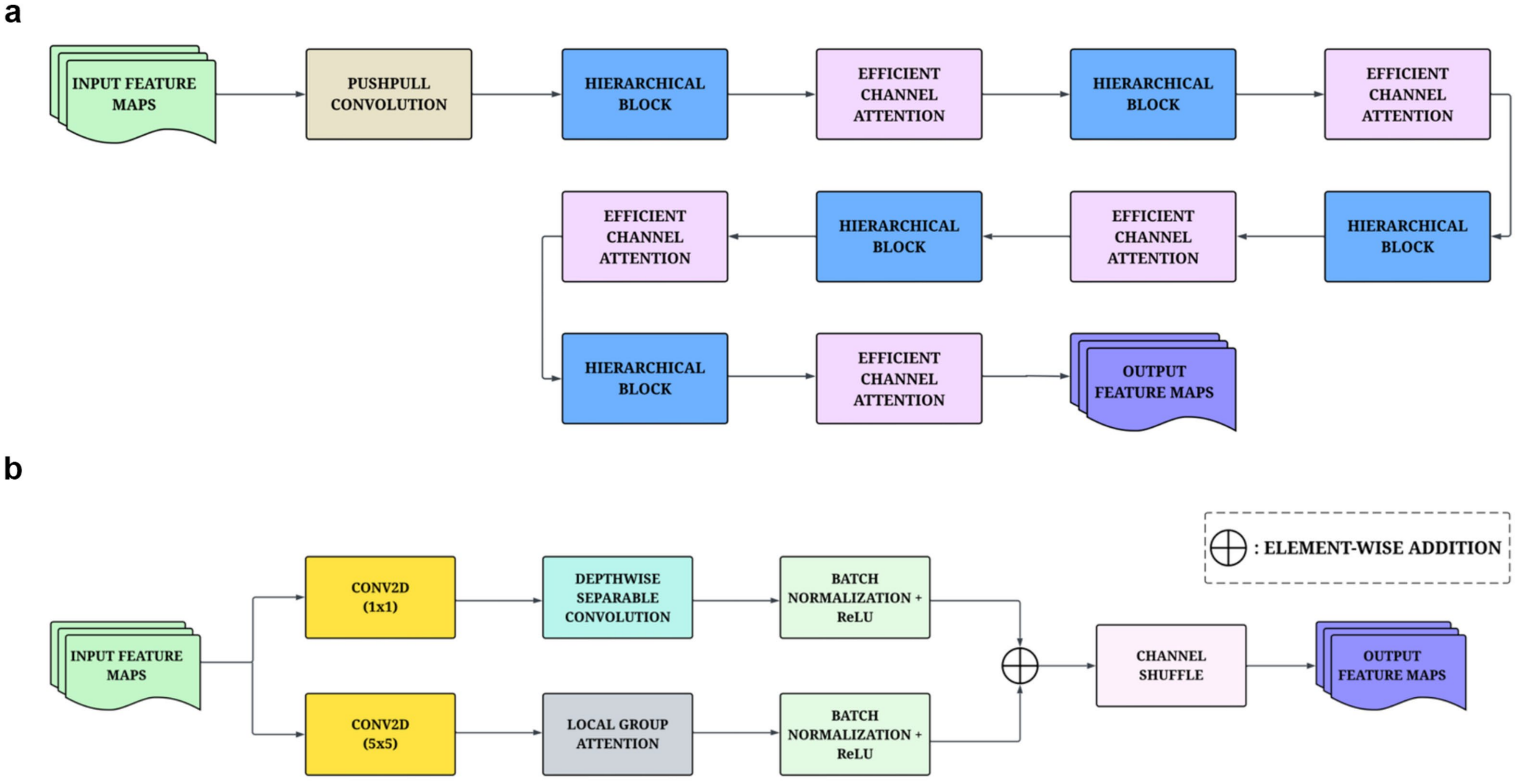
**(a)** The architecture design of the proposed HSGAN track. **(b)** Structural design of the hierarchical block within the HSGAN track.

The outputs from both channels are combined into a residual projection that maintains the same dimensionality. The sum is then spatially shuffled to promote more free exchange of information across the spatial domain where groups exist. In addition, an ECA has been included to indicate the adaptive importance of the data channels based on global context, allowing the model to focus on the most discriminatory features. With push-pull learning rates, hierarchical or multi-level convolution filters, residual operation tasks, adaptive channel importance, and the ECA and LGA modules, the HSGAN generates rich discriminatory feature representations built along with the first track in the dual-path design. Figures 3a,b provide full structural representations of the HSGAN and hierarchical block, respectively.

#### 3.2.1 Efficient Channel attention

The ECA module improves the representational ability of CNNs by fine-tuning channel attention without the need for dimensionality reduction (Wang et al., 2020). In contrast to traditional attention mechanisms that create indirect channel dependencies through reduction operations, ECA computes channel attention directly from the aggregated global features derived from Global Average Pooling (GAP). The central concept of ECA involves effectively modeling local cross-channel interactions through the application of a 1D convolutional operation to the aggregated feature descriptor. Instead of computing attention weights for all the channels of different scales, the module computes the attention weights only for a smaller local neighborhood for each channel. These dimensions of this local neighborhood are determined via consideration of the overall number of channels to find the best convolution kernel dimension to provide an optimal solution. The attention weight of the $i^{\mathrm{th}}$ channel is given by the Equation 5:

$$w_{i}=\sigma (\sum_{j=1}^{k}w_{\mathrm{ji}}y_{\mathrm{ji}})\;\text{where}\;y_{\mathrm{ji}}\in \Omega_{i}^{k}$$ (5)

**Figure 4:**
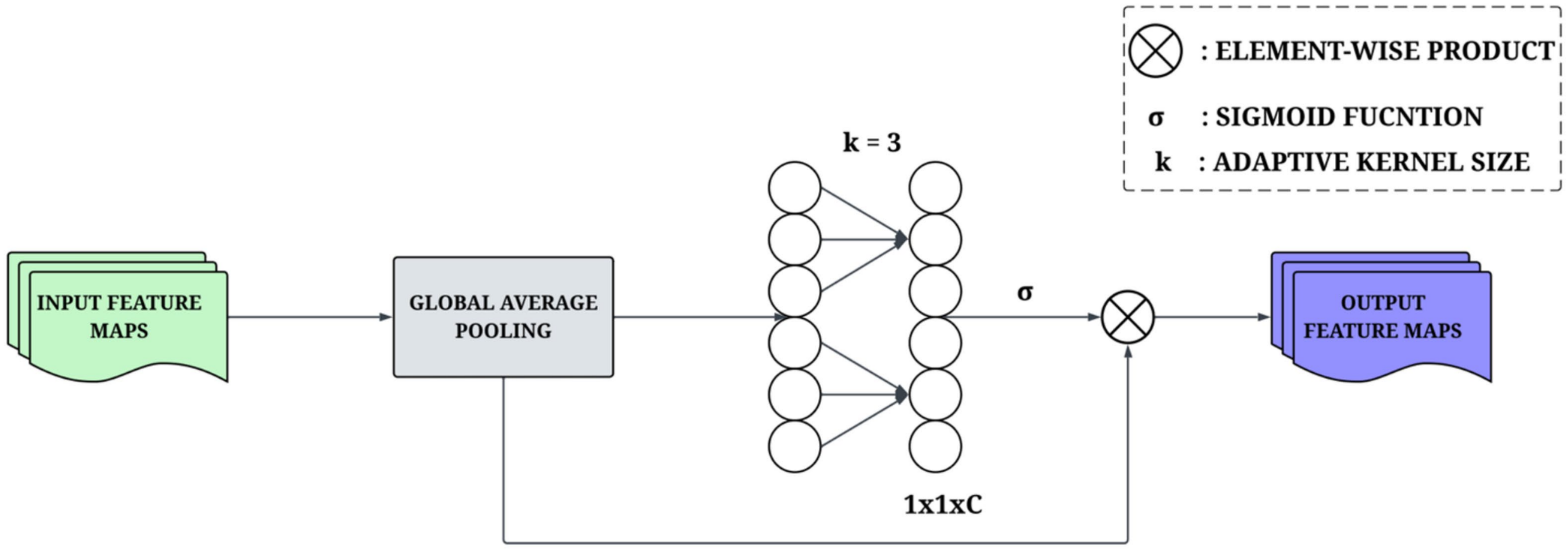
Architectural design of the ECA module for channel-wise feature calibration.

Where, $w_{i}$ represents the weights for each channel, $w_{\mathrm{ji}}$ represents the learnable weights associated with the neighboring channels $y_{\mathrm{ji}}$ , $\Omega_{i}^{k}$ represents the set of k adjacent channels interacting with channel i , and σ(·) is the sigmoid activation function. The formulation of ECA is demonstrated in Equation 6, and it is designed to learn local cross-channel interactions. An adaptive 1D convolution is used to capture that attention across channels, as seen in Figure 4, and the kernel size is automatically derived from the number of channels to allow for adaptive attention.

$$\omega =\sigma ({\text{Conv}}_{k}^{1D}(y))$$ (6)

### 3.3 Multidimensional collaborative attention

The MCA module is introduced as a method to enhance feature representation by simultaneously modeling attention across the channel, height, and width dimensions (Yang et al., 2023). MCA leverages both spatial and channel attention in a unified mode, which provides an effective way to refine features. This design helps provide accurate attenuation of patterns in complex morphological structures. The MCA module consists of three parallel branches, with each focused on a different dimension. Channel attention is focused on discovering the feature channels associated with higher levels of information; height attention identifies important regions along the vertical dimension, and width attention identifies important structures along the horizontal dimension. Every branch uses a two-step process. The squeeze transformation takes and combines feature responses through GAP and standard deviation pooling. This provides access to global and local statistical differences. The excitation transformation offers a simple gating mechanism to enhance meaningful interactions with a low computational burden. For a given input feature map $X\in R^{C\times H\times W}$ the refined outputs of each branch are computed using Equations 7–9.

$$F_{C}^{\prime }=\sigma (W_{C}\cdot T_{\mathrm{EX}}(T_{\mathrm{SQ}}(X_{C})))\cdot X_{C}$$ (7)

$$F_{H}^{\prime }=\sigma (W_{H}\cdot T_{\mathrm{EX}}(T_{\mathrm{SQ}}(X_{H})))\cdot X_{H}$$ (8)

$$F_{W}^{\prime }=\sigma (W_{W}\cdot T_{\mathrm{EX}}(T_{\mathrm{SQ}}(X_{W})))\cdot X_{W}$$ (9)

Where $T_{\mathrm{SQ}}$ denotes the squeeze transformation that extracts meaningful feature statistics, $T_{\mathrm{EX}}$ is the excitation transformation that performs selective enhancement, and $W_{x},W_{H},W_{W}$ are the learnable parameters. The final output is obtained by combining the three branches given by Equation 10:

$$F^{\prime \prime }=\frac{1}{3}(F_{C}^{\prime }+F_{H}^{\prime }+F_{W}^{\prime })$$ (10)

**Figure 5:**
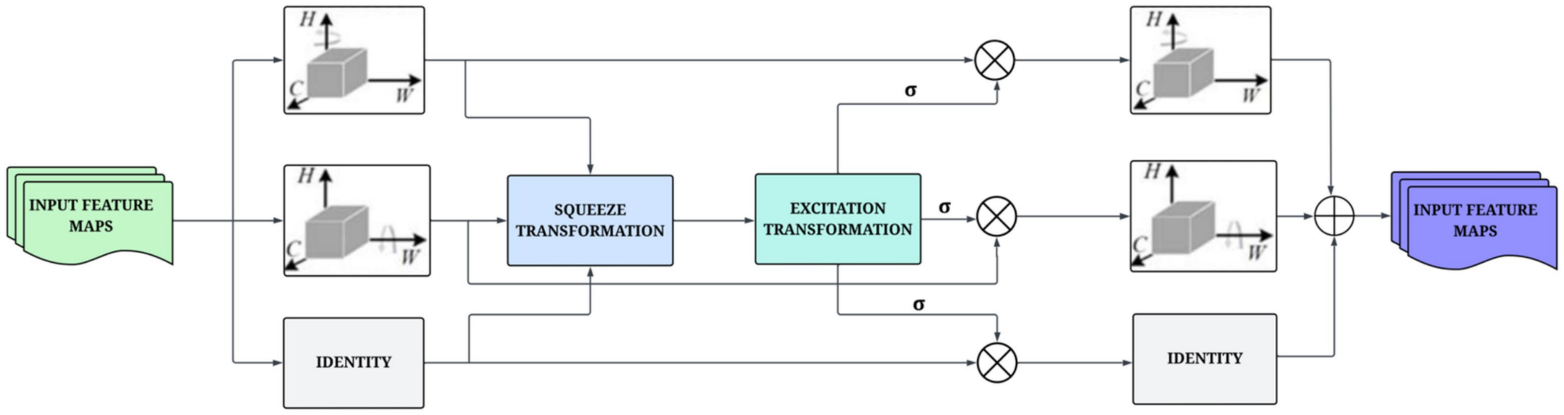
Architectural design of the MCA module that jointly models spatial and channel-wise dependencies for refined feature representation.

This collaborative process helps the model understand the complex structural differences in the nutrient-deficient leaf images. Unlike widely used attention mechanisms, the MCA helps focus exclusively on channel-wise recalibration, providing more expressive feature representation. It reduces the risk of suppressing subtle informative patterns, often overlooked, by jointly modeling spatial and channel correlations. This enhances the model’s sensitivity to fine-grained nutrient deficiency indicators. Figure 5 presents the overview of the architecture of the MCA module.

### 3.4 Classification

The final stage of the proposed framework integrates significant characteristics obtained from both tracks. The representation of features of each mechanism is accomplished through concatenation. The global features extracted from track one are combined with the contextual features derived from track two to create a single, unified representation. To further enrich dependencies between channels and improve the combined feature maps, the MCA is added to model inter-channel relationships. This architecture also implements SPP, which supports the combination of spatial information at multiple levels, allowing for the collection of detailed local information and facilitating the generalization needed to represent more collective patterns of information. The overall design of the architecture effectively enhances the response of the model to complex invariants in spatial information within input images. Subsequently, the features are flattened and sequentially passed through a dropout layer for regularization, followed by dense layers with ReLU activation to represent the number of resulting classes.

## 4 Experiments and results

This section gives an overview of the dataset utilized for this research, the augmentation methods, and the training and testing environment of the experiments. It also presents the hyperparameters considered and their optimal setup. Ablation experiments were carried out to determine the definition of the contribution of the individual tracks and to ascertain the performance of the proposed network.

### 4.1 Dataset description

The research was based on data from coffee leaves in the CoLeaf dataset (Tuesta-Monteza et al., 2023b). The dataset includes 1,006 images of coffee leaves collected from coffee plantations located in San Miguel de las Naranjas and La Palma Central, Jaen, Peru, and the leaf samples were collected systematically from three coffee varieties, CATIMOR, CATURRA, and BORBON, during the second productive stage. The images were provided in the dimensions of 3,000 × 4,000 pixels and recorded in 10 categories of eight nutritional deficiencies: Calcium (Ca), Nitrogen (N), Magnesium (Mg), Potassium (K), Boron (B), Iron (Fe), Manganese (Mn), Phosphorus (P), healthy leaves, and leaves with multiple deficiencies. The provided images were taken with a Canon PowerShot SX50 HS camera in a naturally controlled environment and annotated by an agronomist for leaf symptoms.

### 4.2 Data augmentation

The section discusses the augmentation techniques incorporated in the study. The data were divided into three sets: training, validation, and test. The data were split, with the total number of samples in an approximate 60:20:20 ratio. The augmentation methods that were applied and implemented here augment the variability in the training and validation sets. A horizontal and vertical flip were included to delineate the many orientations of leaves. Rotations of 90°, 180°, and 270° were also addressed in the dataset for variance in position. In addition, photometric augmentations such as random brightness and contrast adjustments were incorporated. Gaussian noise was also added to random samples to improve efficiency against sensor noise and possible image degradation. These methods help reduce overfitting and improve the accuracy, performance, and generalization of the model.

### 4.3 Environmental setup

The study utilized NVIDIA H100 PCIe and Kaggle’s Tesla P100 GPUs for conducting all the experiments. The PyTorch framework was utilized for the implementation of all the models. Adaptive Moment Estimation (ADAM) was incorporated to update network parameters in addition to cosine annealing learning rate for stable convergence in training. The cross-entropy loss function was also implemented to reduce classification errors and improve overall model accuracy.

### 4.4 Hyperparameter tuning

Fine-tuning hyperparameters is an essential part of enhancing the performance of DL models, as it can directly affect the learning mechanism, generalization, and overfitting. The focus of the study was to fine-tune five essential hyperparameters: weight decay, learning rate, optimizer, loss function, and batch size. The weight decay regularization was tested to avoid overfitting and the potential for large weights, which effectively reduces the number of training samples that the model memorizes, within the range of 1e−2 to 1e−6. The learning rate was altered between 1e−2 and 1e−6 for the model to converge consistently. The study also assessed the impact of batch sizes of 8, 16, 32, and 64 to find an optimal value for the efficiency of training and the stochasticity of gradients. The selected hyperparameter ranges were guided by established best practices for CNN-based image classification and preliminary exploratory experiments. This helps ensure stable convergence and prevent overfitting. This constrained search strategy was adopted to maintain experimental consistency across ablation studies and to determine the impact of isolated architectural modifications. Table 1 summarizes the optimal configuration obtained.

**Table 1:** Summary of optimal hyperparameter tuning results.

Hyperparameter	Value range	Optimal
Learning rate	[1e−2, 1e−6]	1e−4
Weight decay	[1e−2, 1e−6]	1e−4
Optimizer	[ADAM, SGD, RMSProp]	ADAM
Loss function	[Cross-entropy loss, Focal loss]	Cross-entropy loss
Batch size	[8, 16, 32, 64]	16

### 4.5 Ablation studies

This section describes the ablation study that was performed to assess the effects of different components on the model’s performance presented in this study. The studies were structured to determine enforceable capabilities to perform better classification on the CoLeaf dataset. The configurations that were studied in this evaluation were (1) MobileNetV3, (2) MobileNetV3 with MCKS, (3) HSGAN, and (4) the proposed network. Each configuration was evaluated using the base performance metrics to provide a better understanding of the contributions to the proposed model.

#### 4.5.1 Analysis of the MobileNetV3 network

**Figure 6:**
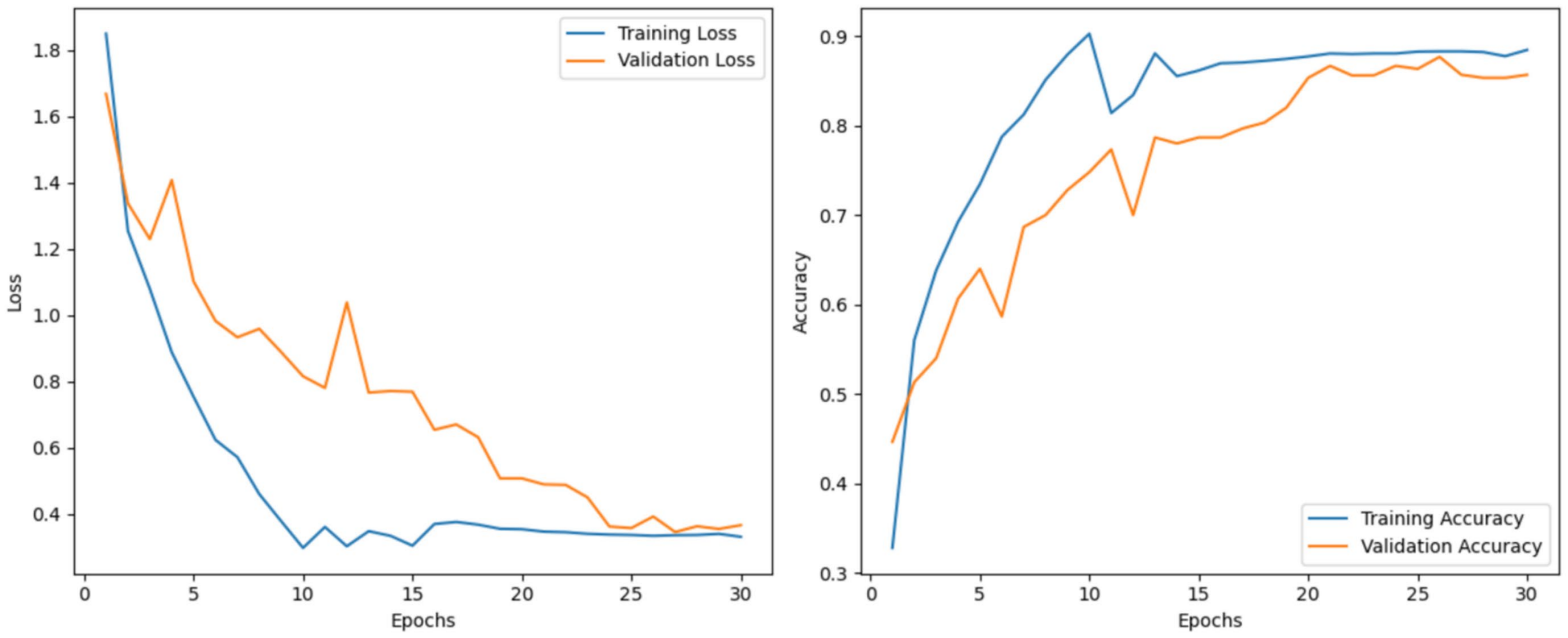
Loss and accuracy curves for training and validation of the MobileNetV3 model.

This section provides an assessment of the MobileNetV3 model on the CoLeaf dataset. MobileNetV3 was utilized as the baseline architecture for feature extraction, followed by a classification layer for predictions at the final stage. Training was conducted utilizing the standard experimental setup with cross-entropy loss and the ADAM optimizer. During testing, MobileNetV3 achieved an accuracy rate of 84.15% with precision, recall, and F1 scores of 80.19, 85.44, and 84.64%, respectively. The performance plots shown in Figure 6 also show consistent convergence and learning patterns from the model.

#### 4.5.2 Analysis of the MobileNetV3 with MCSK block

**Figure 7:**
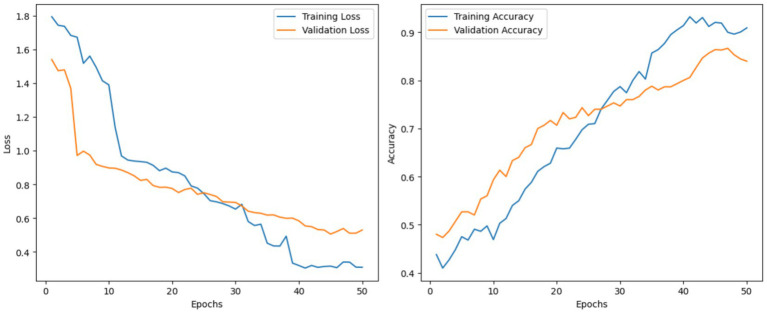
Loss and accuracy curves for training and validation of the MobileNetV3 with the MCSK block.

This section describes the results of adding an MCSK block after the MobileNetV3 model. This MCSK block is added to help the model’s ability to capture significant patterns in leaf disease images and help improve features of the lower- and middle-level features. This resulted in an improvement in accuracy to 87.32% after the MobileNetV3 model had the MCSK block added in. The model achieved a precision of 85.45%, a recall of 86.81%, and an F1 score of 86.98%. Figure 7 illustrates the training and validation accuracy and loss plots of the current ablation study.

#### 4.5.3 Analysis of the HSGAN track

**Figure 8:**
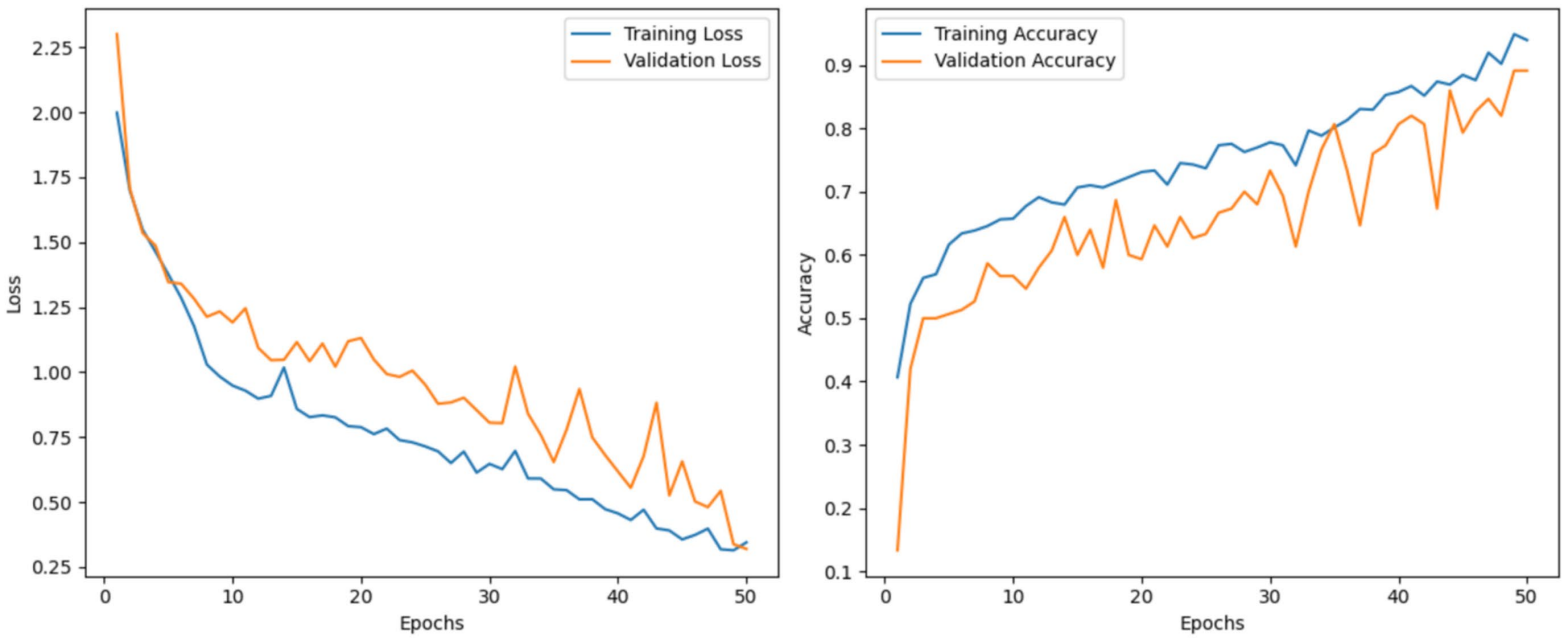
Loss and accuracy curves for training and validation of the HSGAN track.

This section assesses the results from the HSGAN, which is the second track in our dual-track framework. The HSGAN is designed with both convolutional and attention-based methodologies to better capture local context features. In HSGAN, the model starts with a PushPull convolutional module to develop efficacy in the noise. The PushPull is then followed by a hierarchical layout of dual-path blocks, fusing depthwise separable convolutions with LGA to enable the modeling of long-range dependencies with efficiency. The extracted feature maps are then processed through ECA and flattened before classification. The model was trained for 50 epochs with an Adam optimizer and cross-entropy loss. Training and validation accuracy and loss curves are shown in Figure 8. The HSGAN achieved an accuracy of 88.82% on the testing set, with its precision, recall, and F1-scores as 89.12, 89, and 88.84%, respectively. These indicators strongly suggest HSGAN’s prowess in efficiently extracting discriminative features for nutrient deficiency classification.

#### 4.5.4 Analysis of the proposed dual-track architecture

**Figure 9:**
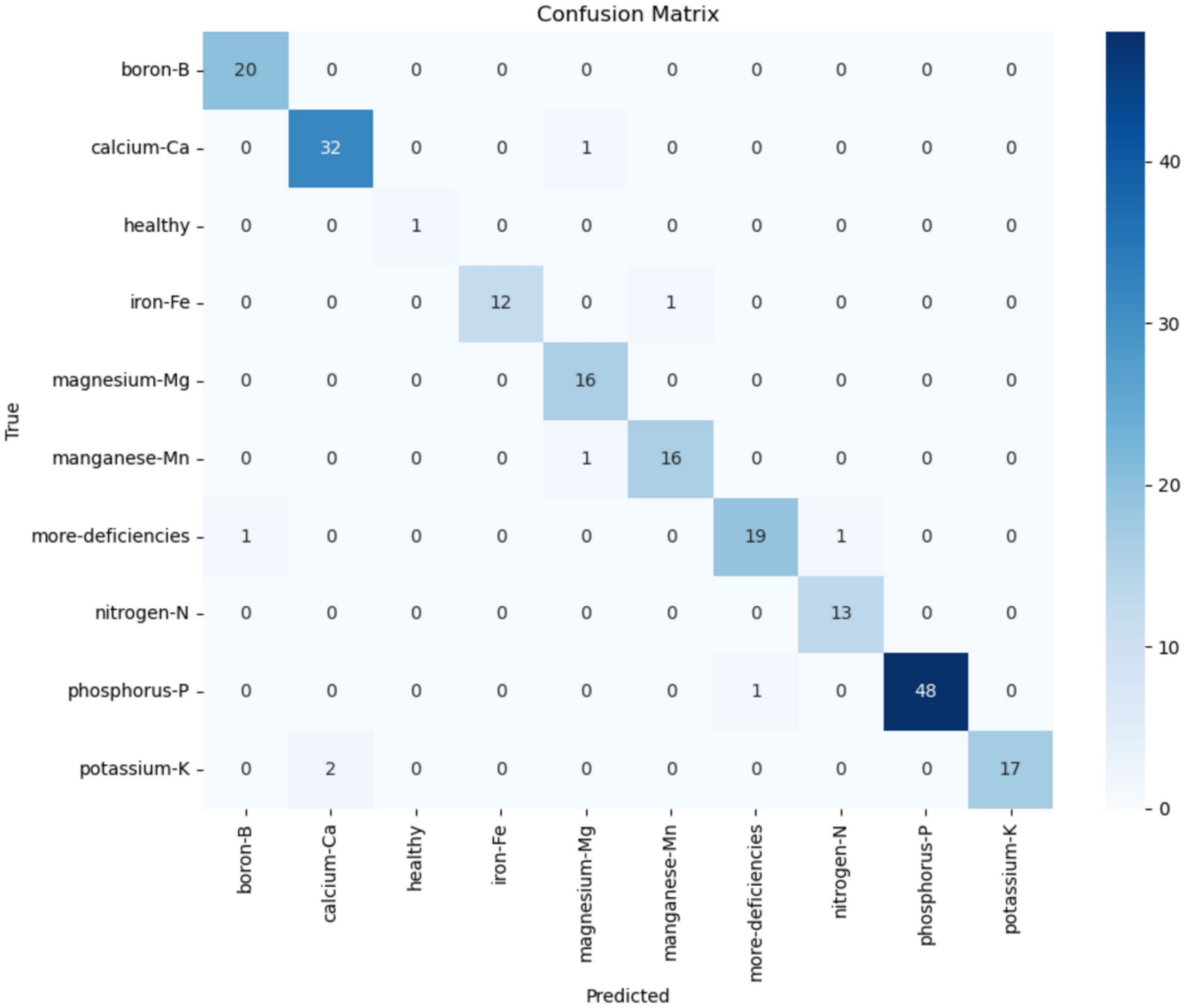
Confusion matrix of the proposed system in classifying nutrient deficiencies in coffee leaf images.

**Figure 10:**
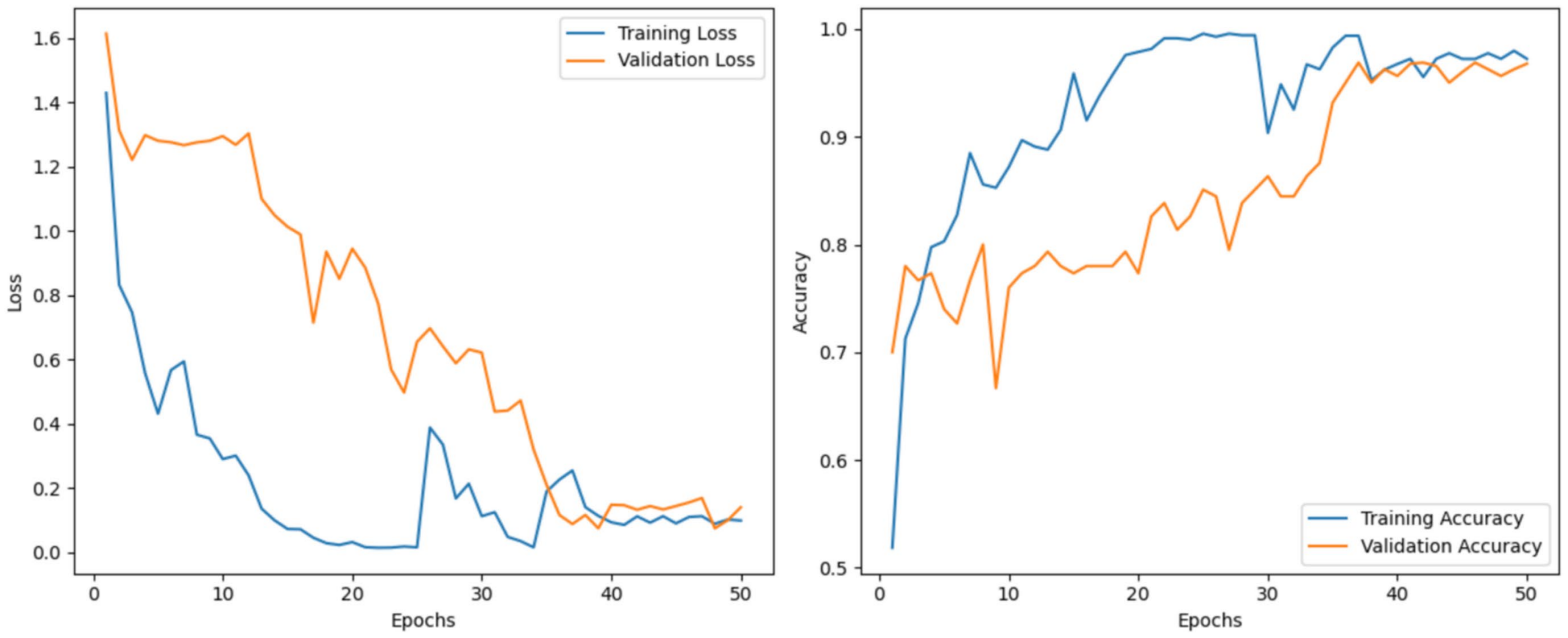
Accuracy and loss curves for training and validation of the proposed architecture.

This section analyses the efficacy of the proposed architecture for nutrient-deficient leaf image classification based on the CoLeaf dataset. The architecture is based on a modified MobileNetV3, which includes an MCSK block in the first track, while the second track includes a custom HSGAN. By utilizing MCSK within the MobileNetV3 architecture, the model achieves a significant increase in its ability to learn and represent leaf textures and vein patterns. The HSGAN track employs push–pull convolution while integrating methods including ECA, LGA, and CS to focus on extracting fine-grained features by modeling channel relationships and spatial correlations, thereby enabling the network to identify subtle color and shape differences related to nutrient deficiencies. Features from both tracks are concatenated to allow for better feature representation, which undergoes further refinement before being passed to the MCA module and SPP layer. This fusion scheme retains multi-scale contextual information and complex local patterns while being invariant to scale, orientation, and leaf structure. Subsequently, the comprehensive feature representation is classified using fully connected layers. Figure 9 illustrates the training and validation curves. The proposed dual-track network showed significant performance when compared to individual configurations, reaching an accuracy of 96.04%. In addition, the achieved precision, recall, and F1 scores were 96.21, 96.04, and 96.04%, respectively. These results highlight the efficiency of global MobileNetV3 features combined with the attention-enhanced local refinement of HSGAN to provide improved accuracy of coffee leaf classification. The associated training and validation accuracy and loss curves are depicted in Figure 10. Beyond the classification metrics, the computation efficiency of the proposed system was also evaluated. The system comprises 70.26 million parameters and requires 8.867 GFLOPs, with a memory footprint of 190.46 MB. Despite the architectural complexity, the proposed architecture achieved an inference time of 21.45 ms per image, demonstrating a near-real-time capability on GPU hardware. This presents a favorable tradeoff between predictive performance and computational cost, making it suitable for decision-support applications. Table 2 summarizes the results of the ablation studies presented. The proposed system was also evaluated under different optimizer choices, with results presented in Table 3.

**Table 2:** Summary of the performance and computational analysis of the ablation studies performed.

Experiment	Number of parameters (in Millions)	FLOPs (in Billions)	Memory usage (in MB)	Precision (in %)	Recall (in %)	F1-score (in %)	Accuracy (in %)	Inference time (in ms)
MobileNetV3	5.48	0.235	21.01	80.19	85.44	84.64	84.15	6.262
MobileNetV3 with MCSK	25.23	3.451	78.45	85.45	86.81	86.98	87.32	10.876
HSGAN	42.78	4.982	95.23	89.12	89.00	88.84	88.82	12.854
Proposed network	70.26	8.867	190.46	96.21	96.04	96.04	96.04	21.449

**Table 3:** Performance analysis of the proposed system based on different optimizer choices.

Optimizers	Precision (in %)	Recall (in %)	F1-score (in %)	Accuracy (in %)
SGD	95.85	95.76	95.81	95.23
RMSProp	95.42	94.00	94.84	95.67
ADAM	96.21	96.04	96.04	96.04

Figure 9 illustrates the confusion matrix evaluated. The confusion matrix demonstrates the proposed model’s strong discriminative power in most classes of nutrient deficiency. It illustrates the ability of the dual-track network to learn global and local representations of coffee leaf features. However, a small number of misclassifications are observed between visually similar nutrient deficiency classes. The most notable misclassification occurs between calcium-Ca and potassium-K. This may be attributed to the biological similarity of their visual symptoms, as both deficiencies often occur as marginal leaf chlorosis. Similarly, a few samples from the iron-Fe and manganese-Mn classes were misclassified, which is possible since both micronutrients exhibit comparable discoloration patterns in coffee leaves. Additionally, a small number of samples from the more-deficiencies class are classified into single-nutrient categories. This result occurs since leaves that are affected by multiple nutrient deficiencies may exhibit dominant visual traits corresponding to the primary class.

Table 4 presents the performance results of the model for each nutrient deficiency class in the dataset. Classes such as healthy, boron-B, iron-Fe, and phosphorous-P achieved strong precision and recall scores, indicating that their visual characteristics are efficiently captured by the proposed model. The recall scores are lower for potassium-K and more deficiencies, highlighting the inherent visual similarity and symptom overlap associated with these classes. Overall, the confusion matrix and class-wise performance metrics present that the proposed system efficiently captures both global structural patterns and fine-grained localized features.

**Table 4:** Class-wise performance metrics obtained from the proposed system.

Class	Precision (in %)	Recall (in %)	F1-score (in %)
Boron-B	95.20	100	97.50
Calcium-Ca	94.10	97.00	95.50
Healthy	100	100	100
Iron-Fe	100	92.30	96.00
Magnesium-Mg	88.90	100	94.10
Manganese-Mn	94.10	94.10	94.10
More-Deficiencies	95.00	90.05	92.70
Nitrogen-N	92.90	100	96.30
Phosphorous-P	100	98.00	99.00
Potassium-K	100	89.50	94.50

### 4.6 Cross-validation

To evaluate the proposed system’s generalization capability, a 5-fold cross-validation strategy was employed. The complete dataset was partitioned into five mutually exclusive and approximately equal subsets. During each iteration, four folds were used for training, while the remaining folds were reserved for validation. This process was repeated five times to ensure that each subset serves as the validation set exactly once. This helps reduce the risk of overfitting and provides a reliable estimate of model performance of the model’s performance across different data partitions. The detailed cross-validation results are presented in Table 5. The model achieved consistent performance across all folds, demonstrating the stability of the proposed model and ensuring the results are not biased toward any particular subset of the dataset.

**Table 5:** Summary of 5-fold cross-validation.

Fold	Precision (in %)	Recall (in %)	F1-score (in %)	Accuracy (in %)
1	94.95	94.86	94.78	94.93
2	94.82	94.78	94.81	94.87
3	94.94	94.89	94.91	94.97
4	94.89	94.80	98.85	94.90
5	94.95	94.88	94.90	94.95

## 5 Discussion

This section offers a detailed assessment of the proposed system. Firstly, it presents the comparative analysis with other state-of-the-art DL models and prior research. Subsequently, the section discusses the limitations of the study and outlines potential directions for future work.

### 5.1 Comparison with state-of-the-art models

This section presents the analysis of the proposed dual-track network, comparing it with various CNN and transformer-based models for classifying nutrient deficiencies in coffee leaves. Among the CNN architectures that were explored, MobileNetV3 provided the highest performance with an accuracy of 84.15%. After MobileNetV3, DenseNet also performed well with an accuracy of 77.56%. EfficientNetB4 achieved an accuracy of 73.25%. The study also included some of the transformer-based models, including the Swin Transformer, which achieved an accuracy of 65.65%. The proposed model achieved competitive performance with an accuracy of 96.04%, which exceeded all predicted baseline models. Additionally, it showed a precision, recall, and F1 score all greater than 96%. These results suggest that the proposed method effectively modeled the broader structure and sufficiently captured detailed local features of nutrient-deficient leaf images.

Additionally, inference efficiency was also analyzed to assess the proposed system’s performance with established state-of-the-art networks. The CNN models, such as ShuffleNet and MobileNetV3, exhibit low inference latency due to their compact architectures, whereas transformer-based networks incur high inference times despite moderate performance. The proposed network consists of 70.26 million parameters and outputs a 21.45 ms inference speed. This is significantly lower than most transformer-based models and deep CNN networks such as VGG16 and VGG19. This highlights a favorable trade-off between accuracy and inference efficiency compared to established baseline networks present in existing literature. Table 6 provides the performance metrics for each of those models, showing the proposed model’s performance along with many CNN and transformer-based models.

**Table 6:** Comparison of the proposed system with state-of-the-art models.

Model	Number of parameters (in millions)	Precision (in %)	Recall (in %)	F1 score (in %)	Accuracy (in %)	Inference time (in ms)
EfficientNet B0	7.48	60.31	60.19	60.25	60.41	3.45
GhostNet	6.21	64.26	60.90	61.92	60.90	4.42
ShuffleNet	5.34	64.72	62.51	63.59	61.73	0.89
ViT	86	65.66	63.33	64.34	61.77	179.53
EfficientNetB2	6.20	64.12	61.78	62.22	61.84	17.95
Pyramid vision transformer	29.76	62.23	62.10	62.20	62.15	150.50
DeiT	87	61.23	64.80	62.96	62.95	45.53
Swin transformer	29.30	65.81	65.20	65.33	65.65	37.56
XceptionNet	22.85	66.16	67.22	66.68	66.67	35.70
ResNet50	26.58	66.98	67.14	67.05	66.90	5.61
EfficientNetB4	7.8	72.34	73.76	73.04	73.25	35.44
VGG16	138	74.90	75.04	74.89	75.28	234.42
VGG19	143	75.09	75.18	75.15	76.22	276.39
InceptionV3	23.62	76.76	76.97	76.75	77.06	39.21
DenseNet	8.03	78.59	76.43	77.56	77.56	23.34
MobileNetV3	5.48	80.19	85.44	84.64	84.15	6.262
Proposed network	70.26	96.21	96.04	96.04	96.04	21.449

### 5.2 Comparison with existing works

Table 7 provides a comprehensive comparison of the proposed network to other methods that were previously utilized with the CoLeaf dataset. The CoLeaf dataset has only recently been published, which binds the number of studies available for direct comparison. Previous works using DL methods such as transfer learning with ResNet50, tailored CNN networks, and hybrid DenseNet-CNN networks all aimed at the classification of nutrient-deficient coffee leaves. The DenseNet with the custom CNN model had significant performance with an accuracy of 95.64%. The proposed dual-track network shows higher accuracy, precision, recall, and F1 scores than the other existing research on the CoLeaf dataset. The results demonstrate the capability of the proposed architecture to effectively capture both fine-grained local and global features in nutrient-deficient coffee leaves.

**Table 7:** Comparative analysis of the performance of the proposed system on the CoLeaf dataset.

S. No	Source	Method	Accuracy (in %)
1.	Tuesta-Monteza et al. (2023a)	ResNet50	87.75
2.	Bera et al. (2024)	Custom CNN	90.54
3.	Sai Charan et al. (2025)	DenseNet with custom CNN	95.64
4.	Proposed system	MobileNetV3 with custom CNN	96.04

### 5.3 Performance evaluation of external datasets

To assess the generalizability of the proposed network, its performance was evaluated on two additional external datasets. The results demonstrate the efficacy and adaptability of the model to new data, emphasizing its potential applicability in diverse agricultural and research settings.

#### 5.3.1 Performance on banana nutrient deficiency dataset

The proposed network was assessed on the publicly available banana nutrient deficiency dataset sourced from Mendeley Data (Sunitha, 2022). The dataset consists of high-resolution images captured using mobile cameras collected across multiple districts of Karnataka. It comprises 3,000 raw images, including eight nutrient deficiency classes, such as boron, calcium, potassium, iron, magnesium, manganese, sulfur, and zinc. The proposed system achieved an accuracy of 90.83%, with precision, recall, and F1 scores of 90.45, 90.16, and 90.28%, respectively. This highlights the model’s generalization ability beyond the primary coffee dataset.

#### 5.3.2 Performance on rice nutrient deficiency dataset

The proposed system was also evaluated on the publicly available rice nutrient deficiency images sourced from the Kaggle platform (Premi, 2024). The dataset includes a total of 1,155 rice leaf images categorized according to different nutrient deficiency types. It consists of 439 samples exhibiting nitrogen (N) deficiency, 333 samples with phosphorous (P) deficiency, and 383 samples representing potassium (K) deficiency. The proposed network achieved an accuracy of 95.84%, with a score of 95.62% precision, 95.38% recall, and 95.50% F1 score. These results further demonstrate the model’s efficacy in generalization capability across different crop species and nutrient deficiency scenarios. Table 8 summarizes the performance of the proposed network across different nutrient deficiency datasets, showcasing its generalizability to diverse agricultural cases.

**Table 8:** Performance comparison of the proposed architecture across external datasets.

Dataset	Accuracy (in %)	Precision (in %)	Recall (in %)	F1-score (in %)
Banana (Sunitha, 2022)	90.83	90.45	90.16	90.28
Rice (Premi, 2024)	95.84	95.62	95.38	95.50
Coffee (Tuesta-Monteza et al., 2023b)	96.04	96.21	96.04	96.04

### 5.4 Limitations and future work

This section outlines the limitations of the proposed model and the implications for future research.

1. While the proposed model demonstrates improved classification accuracy, it has yet to be evaluated real-time field setting. Real-world coffee farms face challenges such as overlapping leaves, insect damage, and light/weather conditions, many of which impact the appearance of symptoms. While increased variation in training sets adds to the uniqueness of the training process, it cannot capture the entire range of phenomena. The model’s performance will improve with more varied environmental data based on geography and conditions.
2. The research does not consider the changes over time in nutritional deficiencies, since the classification relies on static images of leaves. Symptoms of deficiency often develop slowly. This slow development can make it challenging to identify early signs that may be confused with temporary environmental stressors. Integrating sequential image data or longitudinal field observations could improve the system’s capacity to monitor progression and facilitate earlier, more accurate interventions.
3. Although the CoLeaf Dataset serves as an effective benchmark, it does not encompass the full spectrum of nutrient deficiencies for every type of crop and development stage, as well as the environmental conditions in which the crops are grown, and may not fully reflect every form of nutrient deficiency across all classes. Furthermore, there may be less training information for certain classes of deficiency compared with others, which will limit the ability of a model to generalize to that class with respect to less represented categories. Improving the CoLeaf Dataset will enhance the overall accuracy and scalability of the model if equal amounts of training information are available across all classes and if more classes of nutrient deficiencies, in addition to multi-crop deficiency classes, are added.
4. Since the experiments were conducted on a single dataset collected from a specific geographic region, the model’s effectiveness across broader environmental and geographic conditions has not been empirically tested. Future work should include cross-dataset evaluation and in-field data collection from diverse cultivation regions to assess generalization under different climatic, illumination, and environmental factors.
5. Future research should investigate uncertainty-aware prediction and confidence calibration methodologies to improve the model’s interpretability. Approaches such as temperature scaling, Bayesian DL, or Monte Carlo dropout can be incorporated to quantify predictive uncertainty, particularly for visually similar nutrient deficiencies. This allows risk-aware decision support by allowing agronomists to identify low-confidence predictions and aid with targeted field verification.

## 6 Conclusion

Bean growth and yield can be substantially affected by nutritional deficiencies in coffee plants. Therefore, accurately identifying and diagnosing these deficiencies is essential for improving overall crop quality and productivity. Traditional assessment methods rely heavily on expert judgment, making them time-consuming and potentially influenced by subjective bias and assessment timing. These limitations demonstrate the importance of automated, scalable, and reliable solutions within agricultural settings. This study presents a dual-track deep learning architecture for classifying nutrient deficiencies in coffee leaves. The proposed model integrates a modified MobileNetV3 enhanced with MCSK blocks to capture global structural patterns, while an HSGAN-based track focuses on extracting fine-grained local features. Feature representations from both tracks are fused and refined using an MCA module that jointly models spatial and channel dependencies, followed by SPP for multi-scale feature aggregation. This design improves sensitivity to subtle visual indicators of nutrient stress while maintaining computational efficiency. Experimental results on the CoLeaf dataset demonstrate the effectiveness of the proposed approach, achieving 96.04% accuracy along with precision, recall, and F1 scores exceeding 96%.

## Data Availability

Publicly available datasets were analyzed in this study. This data can be found here: https://data.mendeley.com/datasets/brfgw46wzb/1.
